# In Vitro Antimicrobial Activity of Frankincense Oils from *Boswellia sacra* Grown in Different Locations of the Dhofar Region (Oman)

**DOI:** 10.3390/antibiotics9040195

**Published:** 2020-04-20

**Authors:** Vita Di Stefano, Domenico Schillaci, Maria Grazia Cusimano, Mohammed Rishan, Luay Rashan

**Affiliations:** 1 Department of Science and Technology Biological, Chemical and Pharmaceutical (STEBICEF) Università degli Studi di Palermo, Via Archirafi, 32, 90123 Palermo, Italy; vita.distefano@unipa.it (V.D.S.); mariagrazia.cusimano@unipa.it (M.G.C.); 2 Frankincense and Biodiversity Lab, Dhofar University, 2509 Salalah, Oman; mrishan@du.edu.om (M.R.); lrashan@du.edu.om (L.R.)

**Keywords:** *Boswellia sacra*, frankincense essential oil, GC/MS analysis, antimicrobial activity, *Staphylococcus aureus*, *Pseudomonas aeruginosa*, *Propionibacterium acnes*, *Candida albicans*, *Malassezia furfur*

## Abstract

Frankincense essential oils from *Boswellia sacra* have been commonly used to treat microbial infections from as early as the 11th century. The main feature of the plant is its gum resin, from which it is possible to obtain essential oils. In the present study, we focused on the comparative study of the oils extracted from the resins of three different *Boswellia sacra* cultivars (Najdi, Sahli and Houjri). From each of frankincense resin three successive essential oil samples (Grade 1, Grade 2, Grade 3) were obtained. Houjri gum resin gave the lowest percentage (5%) of total essential oil content but showed the maximum number of volatile components in all three grades. Najdi Grade 2 essential oil showed a minimum inhibitory concentration (MIC) of 52 mg/mL toward relevant pathogens *Staphylococcus aureus* and *Pseudomonas* *aeruginosa*, and samples from Grade 2 of Sahily and Houjiri were particularly active against a dermatological strain *Propionibacterium acnes*, displaying MIC values of 0.264 and 0.66 mg/mL, respectively. Data obtained from in vitro studies showed that all essential oils had a significant antifungal effect against *Candida albicans* and *Malassezia furfur*, showing MIC values ranging from 54.56 to 0.246 mg/mL. This work aims to increase the number of substances available in the fight against pathogens and to combat the phenomenon of antibiotic resistance, encouraging the use of alternative resources, especially in non-clinical settings (farms, food processing, etc.).

## 1. Introduction

The *Boswellia* genus of plants includes about twenty species distributed in northeastern coastal areas of Africa, the Arabic peninsula and the Indian subcontinent. *Boswellia sacra*, a small tree of up to 5 m of height with papery peeling bark and densely tangled branches with leaves clustered at the ends, is indigenous to the southern parts of Oman in the Dhofar region, and is also cultivated in other parts of Oman.

*Boswellia sacra* is well known for its oleo-gum resin named frankincense or olibanum, which is usually harvested from deep incisions made into the tree trunk. Generally, in almost all of the cultures where frankincense is traded, it is used for fragrance and fumigating objects used specifically for a religious purpose. In traditional medicine, frankincense has a unique place amongst remedies for the treatment of many disorders (dermatological, gastric, hepatic, rheumatoid arthritis, etc.) [1].

Investigation of the pharmacological activities of essential oils of different *Boswellia* ssp. resins reveals a wide range of medicinal uses. In particular, *B. sacra* has been used as an analgesic, antioxidant, cardio protective substance and an anti-inflammatory [2,3,4]. Frankincense essential oils have been commonly used to treat microbial infections from as early as the 11th century, and some authors have studied the effect of frankincense on urinary tract infections [5]. Resin essential oils have an antimicrobial activity against important human pathogens, both bacterial and fungal organisms, such as *Staphylococcus aureus*, *Escherichia coli*, *Proteus vulgaris* and *Candida albicans* [6,7]. *B. sacra* oil also shows significant activity on *Aspergillus parasiticus* and *Aspergillus flavus* growth, and consequently suppresses aflatoxin production [8].

There are various compounds detected in *Boswellia* species based on several factors, which include color, purity, aroma, clump size, age of the tree, season of harvest and the geographical location of the plant source. The essential oil of *B. sacra* contains a high proportion of monoterpenes (97.3%). The common compounds include α- and β-pinene, limonene, myrcene, linalool and others.

Başer [9] claimed that octyl acetate (39.9%) was the main constituent, followed by 1-octanol (11.9%). Al-Harrasi [10] found limonene (33.5%) and (*E*)-β-ocimene (32.2%) to be the predominant compounds in olibanum from *B. sacra*. Camarda et al. [6] also reported limonene to be the dominant substance, albeit at half the abundance found by Al-Harrasi (18.2%). Furthermore, Camarda identified α-pinene as the second most abundant substance (15.1%). Al-Saidi identified α-pinene as the main volatile component, followed by octyl acetate (13.4%) [11].

**Figure 1:**
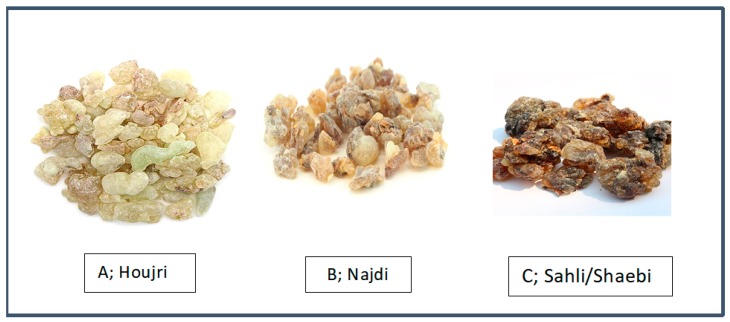
Omani commercially available *B. sacra* resin samples: (**A**) Houjri, (**B**) Najdi, and (**C**) Sahli/Shaebi.

Omani resin is commercially available in *B. sacra* different accessions, under the local names Houjri, Najdi, and Sahli or Shaebi (Figure 1), based on different geographic locations in Dhofar from where the resins are harvested. Houjri, the first grade resin, has the lightest color and a larger clump size, is collected from trees growing in the north of the Samhan mountains, and is the most expensive. Najdi, the second resin, has a pale yellow color and is collected from the plateau behind the Dhofar mountains. Finally, Sahli or Shaebi, which also has a darker color, is collected from the valleys and is the cheaper one.

In the present investigation, we report on the comparative study of the constituents of frankincense essential oil from three different cultivars grown in various agro-climatic conditions in the Dhofar region in Southern Oman, on the eastern border with Yemen. In addition, the authors compared in vitro antibacterial and antifungal activities of *B. sacra* resin essential oils, with the goal to find new strategies in the struggle against antibiotic-resistant microorganisms.

## 2. Results

In Table 1, the essential oil yields from three different frankincense gum cultivars, called Najdi, Sahli and Houjri, are shown. The samples were subjected to three successive hydrodistillations (2, 4, 6 h) to obtain three corresponding essential oils that were called Grade 1, Grade 2 and Grade 3. The essential oils from *B. sacra* oleogum resins were obtained in yields of 8.92–12% (*w*/*w*) in Grade 1. Regarding Grade 2 essential oils, Najdi recorded the highest percentage of 2.20% (*w*/*w*), followed by Sahli, of 2.08% (*w*/*w*). Grade 3 showed the lowest yield percentages of 0.40–0.72% (*w*/*w*) of essential oils (Table 1). However, Grade 1 essential oil samples recorded much higher yield values in comparison to values mentioned in the literature [11]. The essential yields of hydrodistilled *B. sacra* oleo gum resin are comparable to a previous study [10].

All the essential oil samples were investigated and compared for the presence of volatile components, see Table 2. The data obtained from gas chromatography–mass spectrometry (GC/MS) analysis of Grade 1 essential oil indicate the presence of more than 30 components. These chemical components were grouped into three categories, namely monoterpenes, diterpenes and sesquiterpenes.

### 2.1. Composition of Resin Essential Oils

In the case of the cultivar Najdi, a total of 23 active components were identified in the Grade 1 essential oil by GC/MS analysis. The highest percentage was reported for α-pinene, followed by δ-3-carene, camphene and β-pinene (79.59%, 9.94%, 3.23%, and 2.39%, respectively). In the Grade 1 essential oil of Sahli, GC/MS data showed that 29 constituents were present in the essential oil. The major components were α-pinene (78.69%), sabinene (7.78%), camphene (2.66%), limonene (2.31%) and β-pinene (2.66%). In the case of Houjri, GC/MS reports 37 volatile components, which is the highest number compared to Najdi and Sahli with α-pinene, followed by sabinene, camphene, β-pinene, δ-3-carene and cis-verbenol (71.09%, 7.63%, 3.00%, 2.17%, 2.16% and 1.85%, respectively). Data are shown in Table 2.

In Grade 2 essential oil, a sensible reduction of monoterpenes was reported in comparison to Grade 1 essential oil. In all cultivars the major components α-pinene, camphene, β-pinene and δ-3-carene decrease, and the sesquiterpens β-elemene and β-eudesmene increase with a successive 4 h of hydrodistillation.

Grade 3 essential oils show a% reduction in monoterpens (α-pinene, camphene, β-pinene and δ-3-carene) in three cultivars, while at the same time the content in % of sesquiterpenes (β-elemene, β-eudesmene, γ-cadinene) is increased. The highest percentage was recorded for α-pinene. Table 2 shows the results.

### 2.2. Antibacterial Activity of Essential Oils

All the essential oils from the different frankincense cultivars were tested against two relevant pathogens, *S. aureus* and *P. aeruginosa*, included in the global priority list of antibiotic-resistant bacteria from WHO/OMS [12], and against some reference strains of dermatological interest: *Staphylococcus epidermidis* ATCC 12228, *Staphylococcus hominis* ATCC 27,844 and *Propionibacterium acnes* ATCC 11827. The results are expressed in terms of minimal inhibitory concentration (MIC) in percentage *v*/*v* or in mg/mL and are reported in Table 3. The results indicate that Grade 2 essential oil of Najdi showed an MIC of 52 mg/mL for *S. aureus* ATCC 6538, *P. aeruginosa* ATCC 9027 and *P. aeruginosa* ATCC15442. The essential oil of Grade 2 Sahli was active against all reference strains of *S.aureus* and *P. aeruginosa*, with MIC values ranging from 440 to 110 mg/mL. It showed the best MIC values, ranging from 6.16 to 0.264 mg/mL, against bacterial strains of dermatological interest *S. epidermidis*, *S. hominis* and *P. acnes*.

### 2.3. Antifungal Activity of Essential Oils

The antifungal activity was determined against *C. albicans* ATCC 10,231 and *Malassezia furfur* ATCC 14521. All tested samples showed an interesting antifungal activity against *C. albicans* and *M. furfur*, with MIC values ranging from 54.56 to 0.240 mg/mL. In particular, Grade 2 essential oil from Najdi and Grade 1 essential oil from Sahli showed MIC values at the lowest tested concentration corresponding to a percentage *v*/*v* of 0.03 (≤0.252 mg/mL) against both fungal pathogens. The data were shown in the Table 4.

## 3. Discussion

Data presented in Table 2 clearly indicate that there are major differences in the composition of the essential oils of three studied cultivars. All three grades of resin sample gave the highest value for α-pinene, similar findings reported by already mentioned authors [11] and a comparable range for α-pinene was noted in different fractions of the essential oil of *B. sacra* [13]. The percentage of monoterpenes and sesquiterpenes reported for the Grade 1 essential oil of Najdi showed similar values as reported for *Boswellia serrata* [14]. However, there is a major difference in the values recorded for α-pinene in this study, which was 79.59%, while only 5.3% was reported for *B. serrata* [15].

The increased time of extraction through hydrodistillation in three cultivars has resulted in a reduction of monoterpenes, while at the same time the content in % of sesquiterpenes is increased. Moreover, on the basis of data collected, it can be stated that the same species of plants behave differently in terms of the yield of active components of essential oils under different environments. The Najdi sample showed a higher percentage for monoterpenes in all three grades compared to the other two frankincense samples.

Concerning the antimicrobial activity, it was interesting to note that both *S. aureus* and *P. aeruginosa* tested strains were susceptible to most of the essential oils obtained from the three different cultivars. As there are not many antimicrobials concurrently active against the above-mentioned pathogens, these results are relevant because there is a pressing demand for effective antimicrobials towards these microorganisms present on the WHO/OMS priority bacteria list. Therefore, tested essential oils could be a source for new anti-staphylococcal and anti-pseudomonal molecules. Grade 2 of Sahli showed a MIC at 0.264 mg/mL for *P. acnes*, an important pathogen of dermatologic relevance. The activity could be explained by a higher concentration of sesquiterpenes in the extracts obtained by subsequent hydrodistillation of 4 h.

A previous article on four essential oils from different *Boswellia* species showed a significant activity against *Candida tropicalis* and *C. albicans* [16]. In our study we found that all tested samples showed an interesting antifungal activity against *C. albicans* and *M. furfur* with MIC values ranging from 27.28 to 0.240 mg/mL. A high percentage of α-pinene in all Grade 1 essential oils (and in general in monoterpenes) could explain the activity against tested fungal pathogens. Our findings could enable the use of frankincense oil blends in many fields, such as starting products for dermatological bacterial and fungal infections treatments.

In conclusion, GC-MS analysis of *B. sacra* essential oil indicates α-pinene as its primary component. α-Pinene has been found to be one of the integral components of many other antimicrobial essential oils, and thus can be inferred as one of the main players in the antimicrobial activity of *B. sacra* essential oils. Indeed, many of the monoterpenoids putatively identified in our study have been previously reported to have a potent broad antimicrobial spectrum activity. Moreover, the occurrence of drug resistance towards an essential oil in a microorganism is very rare because of its multicomponent nature that necessitates modification of numerous targets [17].

## 4. Materials and Methods

### 4.1. Sample Collection

The different *B. sacra* gum resins were purchased from local market, collected over a period from March to May 2016, and compared with authenticated samples preserved in the Herbarium Center of Nizwa University, Oman, under Voucher number UC29.

The sample named Najdi was obtained from a plateau behind the Dhofar Mountains, where a rapid decline in rainfall and moisture was noted in this region. At the same time, temperature variances rise and desert weather changes the climate of the plain, including the southern slopes. Though annual rainfall in the Jebel region ranges from 500–750 mm, precipitation in the Nejd is recorded only in traces. The sample named Sahli was obtained from valleys (Shabi) of the Dhofar region. The normal annual precipitation is around 110 mm but can be from 70 to 360 mm. July to August is usually the rainy period. The sample Houjri is from Jebel Samhan of the Dhofar region. The Jebel hill range forms a distinct agro-climatic region. Thick fogs are detained back through ranges from inner desert areas. Precipitation is predominantly high and it can be from 600 to 700 mm, the highest in the country, which supports an enduring flora cover.

### 4.2. Extraction of Essential Oils

The essential oils from different cultivars of *B. sacra* were obtained through a hydrodistillation method using Clevenger-type apparatus. A total of 250 g of finely grounded frankincense oleogum resin was added to 2500 mL distilled water in a 5000 mL bottom flask placed in a heating mantle. Hydrodistillation was performed under atmospheric pressure at boiling temperature (about 100 °C) using a closed steam jacket. Following two hours of hydrodistillation, a Grade 1 essential oil of each oleogum resin was collected, whereas Grade 2 and Grade 3 essential oils were collected after 4 and 6 h of successive distillation of the same resin sample. Therefore, a total of nine gum resin essential oils were obtained. The resulted essential oils were collected, dried with anhydrous sodium sulfate, and kept in a dark glass bottle at 4° C until analyzed by gas chromatography–mass spectrometry (GC-MS).

### 4.3. GC/MS Analysis

GC/MS analysis of obtained essential oils was carried out by using a Thermo Scientific DSQ II single quadrupole system of an EI (electron ionization) type, employed in full scan. The temperatures of the injector and ion source were 265 °C and 260 °C, respectively. The capillary column was a ZB-WAX (30 m x 0.25 mm i.e., film breadth 0.25 μm, (Phenomenex, Italy)). The temperature of the oven was set and the column temperature started at 50 °C, was raised at 3 °C/min to 250 °C, and after that a second gradient was used to 300 °C at 40 °C per minute, which was then seized for 3 min under isothermal conditions. The flow rate of helium, the carrier gas, was 1 mL/min. A sample of 1μL was inoculated with a split ratio of 1:100. The temperature of the ion source was 260 °C, the temperature of the MSn transfer line was 265 °C and the temperature of the injector was 270 °C. Ionization voltage was 70 eV and the mass range scanned was 35–550 *m*/*z*. Each oil component was identified on the basis of its retention index, relative to C8-C36 n-alkanes, from MS library searches using the NIST and Wiley GC-MS databases, and by comparison with mass spectral data in the literature.

The percent composition of the essential oil was computed by a standardization process from the GC peak areas, supposing identical mass response factor for all components. Triplicate analyses were prepared for each oil sample.

### 4.4. Antimicrobial Activity, Minimum Inhibitory Concentrations (MICs) Determination

The MICs of essential oils for seven reference bacterial strains (two strains of *Staphylococcus aureus* (ATCC 25923; ATCC 6538), two reference strains of *Pseudomonas aeruginosa* (ATCC 15442; ATCC 9027) and *Staphylococcus epidermidis* ATCC 12228, *Staphylococcus hominis* ATCC 27844, and *Propionibacterium acnes* ATCC 11827) were defined by means of an already described technique for a dilution antimicrobial susceptibility test for bacteria that grow aerobically (CLSI), with few amendments [18]. Concisely, 0.1 mL of essential oil was mixed with 0.1mL of culture medium (Mueller Hinton broth, Sigma-Aldrich, Milan, Italy) present in a well of a sterile 96-wells plate, and a 1:2 dilution series with broth medium was performed. A growth control and a negative sterile well control was included for each plate. A bacterial suspension (10 µL) from a suspension in NaCl 0.9% of isolated clusters, preferred from a 18 to 24 h agar plate and attuned to get a turbidity equivalent to a 0.5 McFarland standard, was added into the wells and the final test concentration of bacteria was approximately 5 × 10^6^ CFU/mL bacterial. The 96-well plates were incubated at 37 °C for 24 h and MICs were read by a microplate spectrophotometer (GloMax^®^-Multi Detection System, Promega Italia s.r.l, Milan, Italy) as the lowest concentration of the sample that fully suppresses bacterial growth in the microdilution wells and whose optical density (OD) at 570 nm is comparable to OD values of negative control wells (only medium, without inoculum).

Antifungal activity against *Candida albicans* (ATCC 10231) and *Malassezia furfur* (ATCC 14521) were evaluated in terms of MICs by using a similar micro-method but by employing Sabouraud broth (Sigma-Aldrich, Milan, Italy) or Sabouraud broth with added 2% olive oil in the case of *M. furfur*, as growth media, and with an incubation time of 48 h.

## 5. Conclusions

This work aims to increase the number of substances available in the fight against infectious diseases and to combat the phenomenon of antibiotic resistance by promoting the use of alternative molecules in non-clinical settings (farms, food processing, airborne decontamination, etc.), preserving conventional antibiotics for clinical application in humans [17]. Worthy of further investigation is also a combination of approaches, combining *B. sacra* essential oils and conventional antibiotics against the global priority pathogens list of antibiotic-resistant bacteria.

## Figures and Tables

**Table 1:** Percentage yields of essential oils obtained from three different frankincense gum cultivars called Najdi, Sahli and Houjri, subjected to three successive hydrodistillations.

Hydrodistillation Extract (Time)	Percentage Yield of Essential Oils (% *w*/*w*)		
	Najdi	Sahli	Houjri
Grade 1 (2 h)	9.32	12.0	8.92
Grade 2 (4 h)	2.20	2.08	1.48
Grade 3 (6 h)	0.68	0.40	0.72
Total	12.20	14.48	11.12

**Table 2:** Chemical constituents identified in Grade 1, 2 and 3 (respectively 2, 4 and 6 h hydrodistillation time) oleogum resin essential oils. RI = retention index relative to C8–C36 *n*-alkanes on ZB-WAX column, MS = NIST and Wiley library and the literature, tr. = trace (<0.01%).

Components of Essential Oils			Grade 1 (2 h)			Grade 2 (4 h)			Grade 3 (6 h)		
			Percentage Essential Oil Composition%								
Compound	RT (min.)	RI	Najdi	Sahli	Houjri	Najdi	Sahli	Houjri	Najdi	Sahli	Houjri
n-nonane	6.05	900	−	−	−	−	−	−	−	−	−
tricyclene	6.65	918	0.10	0.16	0.20	0.10	0.21	0.08	0.08	0.11	0.20
α-thujene	6.82	923	0.10	0.37	0.43	0.21	0.93	0.58	0.22	1.28	0.81
α-pinene	7.08	930	79.59	78.69	71.09	77.21	73.31	63.11	64.45	61.82	62.57
camphene	7.61	946	3.23	2.66	3.00	2.95	2.06	1.62	1.58	1.79	1.48
thujadiene	7.74	950	0.08	010	0.24	0.20	0.37	0.08	0.21	0.43	0.21
sabinene	8.41	970	0.71	7.78	7.63	1.05	3.99	2.40	0.42	2.95	2.48
β-pinene	8.58	975	2.39	2.25	2.17	1.62	2.31	1.53	1.58	1.96	1.43
β-mircene	9.02	988	0.35	0.16	0.32	0.23	0.38	0.36	0.36	0.28	0.14
n-decane	9.45	1000	−			−	−	−	−	−	−
δ-3-carene	9.74	1007	9.94	0.41	2.16	5.39	0.60	0.83	5.88	0.50	1.82
p-cymene	10.38	1023	1.55	0.67	0.90	1.56	0.87	1.49	1.09	1.21	2.60
limonene	10.56	1027	1.23	2.31	0.83	1.35	2.88	1.47	1.84	3.53	2.21
eucalyptole	10.68	1030	0.04	0.06	0.14	0.02	0.01	0.16	0.01	tr.	0.04
cis-sabinene hydrate	12.37	1070	tr.	tr.	tr.	tr.	tr.	0.01	0.02	0.04	0.10
terpinolene	12.92	1084	0.07	tr.	tr.	0.05	0.08	0.02	0.30	0.18	tr.
p-cymenene	13.13	1089	tr.	tr.	tr.	tr.	tr.	tr.	tr.	tr.	tr.
linalool	13.48	1097	0.01	0.17	0.19	tr.	0.02	0.12	tr.	0.02	0.12
n-undecane	13.61	1100	−			−	−	−	−	−	−
fenchone	13.93	1108	tr.	0.13	0.17	tr.	tr.	0.33	tr.	tr.	0.17
α-campholenol	14.71	1125	tr.	0.21	0.35	tr.	0.15	0.45	tr.	0.24	0.15
trans-pinocarveol	15.29	1139	0.02	0.15	0.71	0.09	0.70	1.06	0.12	0.45	0.62
cis-verbenol	15.52	1144	tr.	0.80	1.85	0.26	0.61	1.73	0.10	0.50	0.84
pinocarvone	16.25	1160	tr.	tr.	0.02	tr.	tr.	tr.	tr.	tr.	0.04
cis-sabinol	16.69	1170	tr.	tr.	tr.	0.01	0.53	0.08	0.37	1.22	0.06
4-terpineol	17.08	1180	tr.	0.28	0.76	0.01	1.70	1.50	0.16	2.43	2.27
p-cymen-8-ol	17.41	1187	tr.	tr.	0.21	tr.	0.24	0.88	0.01	0.24	0.53
α-terpineol	17.74	1195	0.01	0.18	0.60	0.97	0.90	1.42	1.84	1.09	1.34
n-dodecane	18.07	1200	−			−	−	−	−	−	−
verbenone	18.24	1204	tr.	0.28	0.92	tr.	0.53	2.06	0.15	0.69	1.35
trans-carveol	18.84	1218	tr.	tr.	tr.	tr.	tr.	0.23	0.01	tr.	0.01
bornyl acetate	21.66	1281	0.31	0.49	0.93	0.72	1.01	1.49	1.87	1.50	1.11
thymol	22.24	1294	tr.	tr.	0.48	tr.	tr.	0.63	tr.	tr.	0.18
n-tridecane	22.51	1300				−	−	−	−	−	−
carvacrol	22.77	1306	tr.	tr.	0.11	tr.	tr.	0.19	tr.	tr.	0.07
δ-elemene	24.39	1344	tr.	tr.	0.12	0.17	0.19	0.74	0.32	0.60	0.32
α-copamene	25.580	1371	0.02	0.04	0.08	0.37	0.11	0.22	0.50	0.21	0.25
β-bourbonene	25.90	1379	0.06	0.24	0.09	0.46	0.48	0.87	0.96	1.00	0.33
β-elemene	26.18	1385	0.01	0.44	0.87	1.40	1.18	3.72	4.68	2.79	4.55
n-tetradecane	26.82	1400	−			−	−	−	−	−	−
β-caryophyllene	27.38	1414	0.07	0.28	0.28	0.59	0.76	0.73	0.75	1.48	0.64
α-humulene	28.87	1450	tr.	0.10	0.11	0.41	0.23	0.28	0.54	0.46	0.35
allo-aromadendrene	29.04	1454	tr.	tr.	0.03	tr.	0.10	0.14	0.21	0.16	0.12
γ-muurolene	29.63	1470	tr.	tr.	tr.	0.01	tr.	0.01	0.01	tr.	0.04
β-eudesmene	30.24	1483	0.06	0.39	0.78	1.01	1.18	2.50	3.52	3.07	3.46
azulene	30.53	1490	0.01	0.15	0.30	0.83	0.55	0.90	1.87	1.35	1.19
n-pentadecane	30.94	1500	−			−	−	−	−	−	−
γ-cadinene	31.45	1513	tr.	0.03	tr.	0.62	0.50	0.05	2.71	1.39	0.17
caryophyllene oxide	33,10	1527	tr.	tr.	0.19	tr.	tr.	1.01	0.09	0.21	1.14
n-hexadecane	34,01	1600	−			−	−	−	−	−	−
viridiflorol	35,15	1633	tr.	tr.	tr.	0.12	tr.	0.38	0.40	0.10	0.46
τ-cadinol	36,77	1638	tr.	tr.	0.02	tr.	tr.	tr.	tr.	tr.	0.12
α-eudesmol	37,19	1641	tr.	tr.	0.03	tr.	tr.	0.80	tr.	tr.	0.91

**Table 3:** Antibacterial activity of essential oils expressed in terms of minimum inhibitory concentration (MIC) in %*v*/*v* or mg/mL (between brackets).

Cultivar	Essential Oil	*S. aureus* ATCC 25923	*S. aureus* ATCC 6538	*P. aeruginosa* ATCC 9027	*P. aeruginosa* ATCC 15442	*S. epidermidis* ATCC 12228	*S. hominis* ATCC 27844	*P. acnes* ATCC 11827
Najdi	Grade 1	25	25	>25 (>210)	>25 (>210)	6.2 (52)	25 (210)	>25 (>210)
	Grade 2	25 (210)	6.2 (52)	6.2 (52)	6.2 (52)	50 (420)	>25 (>210)	>25 (>210)
	Grade 3	12.5 (112.5)	12.5 (112.5)	12.5 (112.5)	12.5 (112.5)	25 (225)	>25 (>225)	>25 (>225)
Sahli	Grade 1	25 (210)	12.5 (105)	25 (210)	25 (210)	0.7 (5.88)	25 (210)	25 (210)
	Grade 2	12.5 (110)	25 (220)	50 (440)	12.5 (110)	0.3 (2.64)	0.7 (6.16)	0.03 (0.264)
	Grade 3	25 (210)	25 (210)	>25 (>210)	>25 (>210)	25 (210)	25 (210)	>25 (>210)
Houjri	Grade 1	25 (200)	25 (200)	25 (>200)	25 (>200)	50 (400)	>25 (>200)	>25 (>200)
	Grade 2	25 (237.5)	50 (475)	25 (237.5)	25 (237.5)	1.5 (14.25)	0.3 (2.85)	0.07 (0.66)
	Grade 3	25 (200)	25 (200)	25 (200)	25 (200)	25 (200)	25 (200)	>25 (>200)

**Table 4:** Antifungal activity MIC values in %*v*/*v* and in mg/mL between brackets.

Essential oil	*Candida albicans* ATCC 10231	*Malassezia furfur* ATCC 14521
Grade 1 Najdi	≤0.03 (≤ 0.252)	≤0.03 (≤ 0.252)
Grade 2 Najdi	≤0.03 (≤ 0.252)	≤ 0.03 (≤0.252)
Grade 3 Najdi	≤ 0.03 (≤0.270)	≤0.03 (≤0.270)
Grade 1 Sahli	≤ 0.03 (0.252)	≤0.03 (≤0.252)
Grade 2 Sahli	3.10 (27.28)	≤0.03 (≤ 0.264)
Grade 3 Sahli	1.50 (54.56)	1.50 (54.56)
Grade 1 Houjri	≤0.03 (≤0.240)	≤0.03 (≤0.240)
Grade 2 Houjri	1.50 (14.25)	0.15 (1.425)
Grade 3 Houjri	1.50 (12.60)	0.07 (0.588)
